# Circular RNA SOX5 promotes the proliferation and inhibits the apoptosis of the hepatocellular carcinoma cells by targeting miR-502-5p/synoviolin 1 axis

**DOI:** 10.1080/21655979.2022.2029110

**Published:** 2022-01-20

**Authors:** Yu Cai, Yuanyuan Jia

**Affiliations:** a Department of General Surgery, The First Affiliated Hospital of Xi’an Medical University, Xi’an, Shaanxi, China; b Department of Faculty Development and Teaching Evaluation Office, The First Affiliated Hospital of Xi’an Medical University, Xi’an, Shaanxi, China

**Keywords:** Hepatocellular carcinoma, apoptosis, synoviolin 1, circ-SOX5

## Abstract

We aimed to explore the role of circ-SOX5 in the pathogenesis of hepatocellular carcinoma (HCC). The circRNAs in HCC were screened using the GEO database. RT-qPCR was used to detect mRNA expression. Targeting relationships were confirmed by dual luciferase reporter assay and RNA pull-down assay. CCK-8 and EDU staining were used to measure cell viability and proliferation, respectively. Cell apoptosis was determined using flow cytometry. Protein expression was determined by Western blotting. Circ-SOX5 expression was increased in HCC tissues. Inhibition of circ-SOX5 expression reduced the viability, proliferation, and colony formation, and increased the apoptosis of HCC cells. However, miR-502-5p inhibition or overexpression of synoviolin 1 (SYVN1) can reverse the effects of circ-SOX5 knockdown on proliferation and apoptosis. This study demonstrated that the circ-SOX5/miR-502-5p/SYVN1 axis promotes the development of HCC by regulating cell apoptosis. Therefore, circ-SOX5 may be a potential biomarker of HCC.

## Graphical abstract

**Figure:**
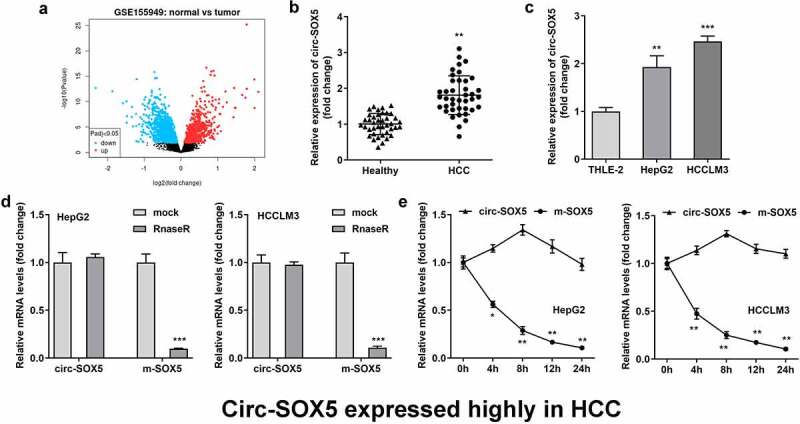

## 1. Introduction

Hepatocellular carcinoma (HCC) is a common malignant tumor that is highly metastatic and aggressive. In 2020, new cases of liver cancer accounted for 4.7% of the new cases of cancer in the whole year and ranks second in cancer-related deaths globally [1,2]. Chinese patients account for approximately half of the total number of HCC cases worldwide [3]. HCC is secretive at an early stage, and most HCC cases are diagnosed at an advanced stage. Moreover, the five-year survival rate after surgery is low [4], and the recurrence and metastasis rates are more than 60% [5]. Thus, identifying a sensitive biomarker for HCC is of vital importance.

Apoptosis is a gene-controlled autonomous cell death system proposed by Kerr in 1972 [6]. It helps eliminate excess cells and injured cells, affect the tissue and organ development, and establish the immune system [7,8]. Apoptosis occurs through two pathways: the death receptor-mediated pathway and the mitochondrial pathway [9]., and both ultimately activate the cysteine protease (caspase) to trigger apoptosis [10]. Apoptosis can also regulate tumors [11]. Tumor radiotherapy and chemotherapy can also be implemented by inducing apoptosis [12]. However, the underlying mechanisms remain unclear.

Circular RNAs (circRNAs) are a class of non-coding RNAs that are widely distributed in various organisms, with a covalently closed loop structure [13]. CircRNAs have no 5′ cap and 3′ poly (A) tail, and cannot be degraded by exonuclease, so are more stable than linear RNA [14]. CircRNAs are usually produced in a non-classical splicing form, which is ubiquitous in gene expression program of human cells [15,16]. Memczak et al. confirmed that circRNAs show a very important regulatory effect on gene expression at the post-transcriptional level [14]. CircRNAs block the inhibitory effect of miRNA on target RNA by binding to microRNAs (miRNAs), thereby regulating the expression level of miRNA target genes [17]. circRNAs have been reported to affect cancers such as gastric cancer [18], lung cancer [19], and liver cancer [20]. In addition, the upregulation of multiple circRNAs in HCC promotes the proliferation and invasion of cancer cells and reduces the occurrence of apoptosis [21–23]. However, very few studies have been conducted to understand the role of circRNAs in cancer and more studies are needed for further clarification.

Therefore, this study aimed to explore circ-SOX5 in HCC and its effect on apoptosis. We hypothesize that circ-SOX5 participated in the HCC progression via regulating the miR-502-5p/SYVN1 axis

## 2. Materials and methods

### 2.1 Patient

Samples were collected from HCC patients (n = 40) as well as healthy volunteers (n = 40) at the First Affiliated Hospital of Xi’an Medical University from 20 October 2019 to 20 October 2020.

The study was approved by the First Affiliated Hospital of Xi’an Medical University. Signed informed consents were obtained from all study participants.

### 2.2 Cell culture and cell transfection

THLE-2, HepG2, and HCCLM3 cells were purchased from the Type Culture Collection of the Chinese Academy of Sciences (Shanghai, China). DMEM with 10% fetal bovine serum, 100 mg/mL of penicillin, and 100 U/mL of streptomycin (Gibco, Waltham, USA) was used to culture cells. The cells were maintained in the presence of 5% CO _2_ at 37°C.

Circ-SOX5, circ-SOX5 small interference RNA (si- circ-SOX5), miR-502-5p inhibitor, SYVN1 (Abiocenter Biotech Co., Ltd.) were transfected into cells using Lipofectamine® 2000 reagent (Invitrogen) at 37°C according to the manufacturer’s protocols. After 48 h of transfection, cells were used for the following experiments.

### 2.3 RT-qPCR

RNA samples were extracted from all the cells using a commercially available kit (Takara, Japan) [24]. Then, cDNA was synthesized and PCR was performed using a Real-Time PCR Detection System (Bio-Rad, USA). The primer sequences used were as follows:

circ-SOX5: F: 5′-TGCTCCAGCAACAGATCCAG-3′, R: 5′-ATAGCTGAAGCCTGGAGGGA-3′; miR-502-5p: F: 5′-CACCTGGGCAAGGATTCA-3′, R: 5′-CTCAACTGGTGTCGTGGAGTC-3′; SYVN1: F: 5′-CTTCGTCAGCCACGCTTATC-3′, R: 5′-CCACGG AGTGCAGCACATAC-3′.

### 2.4 Cell viability assay

After resuspending at 1 × 10^5^ cells/ml, HepG2 and HCCLM3 cells were seeded in 96-well plates at 100 μl/well according to a previous study [25]. CCK8 reagent 10 μl (AmyJet Technology Co., Ltd.) was added to each well and incubated for 4 h at 37°C. A microplate reader (Nanjing DeTie Experimental Equipment Co., Ltd.) was used to measure absorbance at 450 nm.

### 2.5 5-Ethynyl-2′-deoxyuridine assay

HepG2 and HCCLM3 cells were treated with EdU dye and fixed with 4% paraformaldehyde according to a previous study [26]. Cells were stained with 1X Apollo reaction cocktail for 30 min before incubation with Hoechst 33,342. Cells were then observed and images were recorded using a fluorescence microscope (Leica, Germany).

### 2.6 Flow cytometry assay

The apoptosis of HepG2 and HCCLM3 cells was measured using the TransDetect® Annexin V-FITC/PI Kit (FA101-01; TransGen Biotech Co., Ltd.) [27]. Briefly, 5 μl Annexin V-FITC was added to a 6-well plate, and the cells were incubated for 15 min in the dark at room temperature. The apoptosis rates of HCC cells were determined using a NovoCyte Advanteon B4 Flow Cytometer and NovoSampler Q software (Agilent Technologies Co., Ltd.).

### 2.7 Western blot analysis

According to a previous study [28], protein extracts were subjected to 10% SDS gel electrophoresis. The protein extracts were then transferred to a polyvinylidene fluoride membrane (Millipore), and incubated with primary antibodies at 4°C overnight. The next day, the membrane was incubated with the secondary antibodies for two hours at room temperature. Finally, the images were captured using an ECL system (Thermo Fisher Scientific, Inc.).

### 2.8 Transwell assay

According to previous study [29], cell culture Transwell inserts were placed into the 48-well plates to generate the departed upper and lower chambers following 48 h transfection. Besudes, Matrigel was selected to pre-coat the upper of the membrane. The cells in the upper chamber were then cultured for 1 h at 37°C. The membrane was hydrated with FBS 2 h prior. 600 µl of RPMI-1640 supplemented with 10% FBS and 1 × 10^5^ cells/well was placed into the lower and upper chamber separately. Each experiment was repeated 3 times. After 24 h of culturing, a microscope was used to count the numbers of invaded and migrated cells.

### 2.8 Dual luciferase reporter assay

The wild-type (WT) and mutant (MUT) type 3-UTR regions of circ-SOX5 and SYVN1 luciferase reporter vectors were designed and synthesized by Guangzhou RiboBio Co., Ltd according to a previous study [30]. After cultivation for 24 h, the cells were lysed. Luciferase Reporter Assay Kit (K801-200; BioVision Tech Co., Ltd.) was used to analyze luciferase activity 48 h after co-transfection with miR-18b-5p mimic/control and the luciferase reporter vectors. Finally,, luciferase activity was normalized to Renilla luciferase activity.

### 2.9 RNA pull-down

RNA pull-down assays were performed using MagCapture™ RNA Pull Down Assay Kit (Whatman Co., Ltd.) following the manufacturer’s protocols according to a previous study [31]. Proteins were then collected for mass spectrometry analysis.

### 2.10 Statistical analysis

Each experiment was carried out 3 times. All data were calculated using GraphPad Prism and are presented as mean ± SD. Student’s t-test was performed to compare the differences between two groups, the contrast of multiple groups with the analysis of variance (ANOVA) followed by Duncan’s post-hoc test. The starbase online database was used to predict the target miRNA of circ-SOX5, and targetscan online database was used to predict the target gene of miR-502-5p. P < 0.05 suggested a significant difference.

## 3. Results

Circ-SOX5 was significantly up-regulated in the HCC cells. Inhibition of circ-SOX5 increased the apoptosis of tumor cells and reduced the proliferation of tumor cells through miR-502-5p/SYVN1 axis. We demonstrated that circ-SOX5 might be a potential therapeutic target or diagnostic marker for the treatment of HCC.

### 3.1 Circ-SOX5 expressed highly in HCC

**Figure 1.**
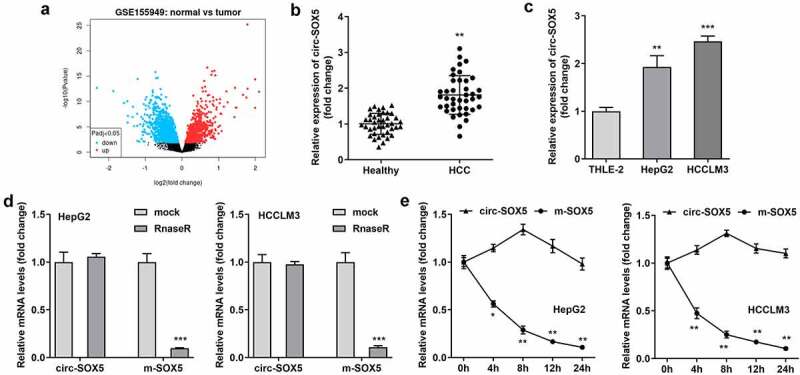
**Circ-SOX5 was up-regulated in HCC**. (a) Volcano plots indicated the differentially expressed circRNAs between HCC and normal samples from the GSE155949 datasets. (b) circ-SOX5 expression in HCC. (c) circ-SOX5 expression in HCC cells. (d) Expression of circ-SOX5 in hcc cells after treatment with RNaseR. (e) Half-life of circ-SOX5 and SOX5 mRNA in HCC cells. **P < 0.01, *** P < 0.001 versus control.

To select circRNAs related to HCC, we analyzed the microarray data of GSE155949 from the GEO database. Compared with normal tissues, 5726 circRNAs were found upregulated in HCC tissues, while 4868 circRNAs were downregulated. The expression of circ-SOX5 in HCC tissues was notably increased (Figure 1(a)). In addition, compared with that in normal controls, circ-SOX5 in the serum of HCC patients was significantly increased (Figure 1(b)). Meanwhile, the expression of circ-SOX5 in HepG2 and HCCLM3 cells was also significantly increased (Figure 1(c)). Figure 1(d) shows that in HepG2 and HCCLM3 cells, compared with circ-SOX5, the expression level of SOX5 mRNA was markedly decreased after RNaseR treatment. Half-life of SOX5 mRNA was only about 4 h, while circ-SOX5 had a half-life of more than 24 h (Figure 1(e)), suggesting more stable RNA expression of circ-SOX5.

### 3.2 Knockdown of circ-SOX5 reduced cell viability, proliferation as well as colony formation, while increased apoptosis of HCC cells

**Figure 2.**
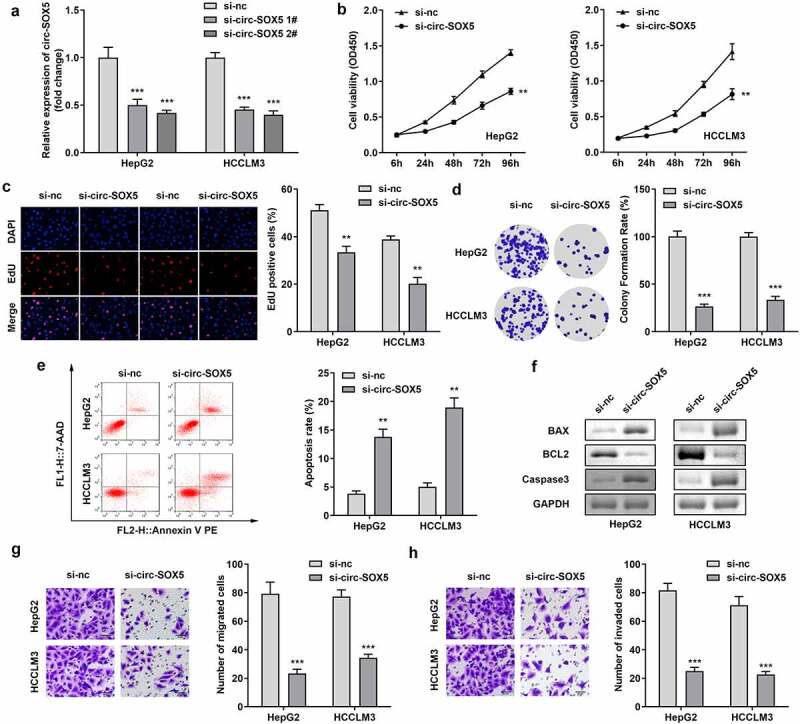
**Circ-SOX5 knockdown reduced cell viability, proliferation as well as colony formation, increased apoptosis of HCC cells**. (a) Expression of circ-SOX5 in HCC cells. (b) HCC cells viability. (c) HCC cells proliferation. (d) Clone formation of HCC cells. (e) The apoptosis of HCC cells. (f) The protein expression of BAX, BCL2 as well as Caspase3. (g) The migration of the HCC cells. (h) The invasion of the HCC cells. **P < 0.01, *** P < 0.001 versus control.

circ-SOX5 in HepG2 and HCCLM3 cells was notably decreased after knocking down, indicating successful transfection. Moreover, si-circ-SOX5 2 # was more remarkable, which was used in subsequent experiments (Figure 2(a)). Knockdown of circ-SOX5 significantly inhibited cell viability, proliferation, and clone formation of HCC cells (Figure 2(c-d)). The apoptosis was significantly increased after circ-SOX5 knockdown (Figure 2(e)). Moreover, expression of proteins BAX was well as caspase-3 while decreased BCL2 was decreased (figure 2(f)). Additionally, Knockdown of circ-SOX5 significantly decreased the cell migration and invasion of HCC cells (Figure 2(g-h)).

### 3.3 Circ-SOX5 directly targeted miR-502-5p

**Figure 3.**
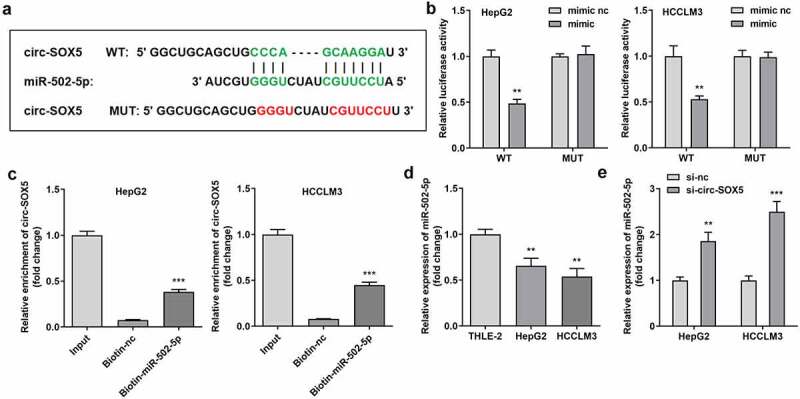
**Circ-SOX5 sponged miR-502-5p**. (a) The binding sites between circ-SOX5 and miR-502-5p. (b) Luciferase activity of HepG2 and HCCLM3 cells. (c) Interaction between circ-SOX5 as well as miR-502-5p. (d) miR-502-5p expression in HepG2 and HCCLM3 cells. (e) miR-502-5p expression in cells with circ-SOX5 knockdown. **P < 0.01, *** P < 0.001 versus control.

We looked for the potential target miRNAs of circ-SOX5. Figure 3(a) shows the binding sites of miR-502-5p as well as circ-SOX5, verified by dual luciferase reporter assay and RNA pull-down assay (Figure 3(b-c)). miR-502-5p expression was significantly reduced in HCC cells (Figure 3(d)). Moreover, circ-SOX5 knockdown significantly upregulated miR-502-5p expression (Figure 3(e)).

### 3.4 miR-502-5p directly targeted SYVN1

**Figure 4.**
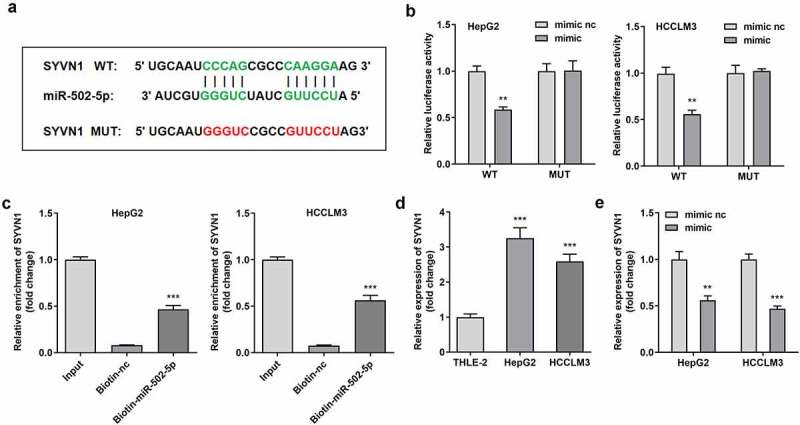
**MiR-502-5p bound with SYVN1**. (a)Binding sites of miR-502-5p as well as SYVN1. (b) Luciferase activity of HepG2 and HCCLM3 cells. (c) Interaction between SYVN1 as well as miR-502-5p. (d) SYVN1 expression in HepG2 and HCCLM3 cells. (e) SYVN1 expression in cells with miR-502-5p overexpression. **P < 0.01, *** P < 0.001 versus control.

In order to determine the regulatory axis, including circ-SOX5 and miR-502-5p, we investigated target gene of miR-502-5p, and found SYVN1 (Figure 4(a)), which was confirmed by RNA pull-down assay and dual luciferase reporter assay (Figure 4(b- c)). Compared with normal liver cells, expression of SYVN1 in liver tumor cells was significantly increased. Additionally, miR-502-5p overexpression significantly reduced the expression of SYVN1 (Figure 4(e)).

### 3.5 Inhibition of miR-502-5p and overexpression of SYVN1 reversed the effects of circ-SOX5 knockdown on cell viability and apoptosis

**Figure 5.**
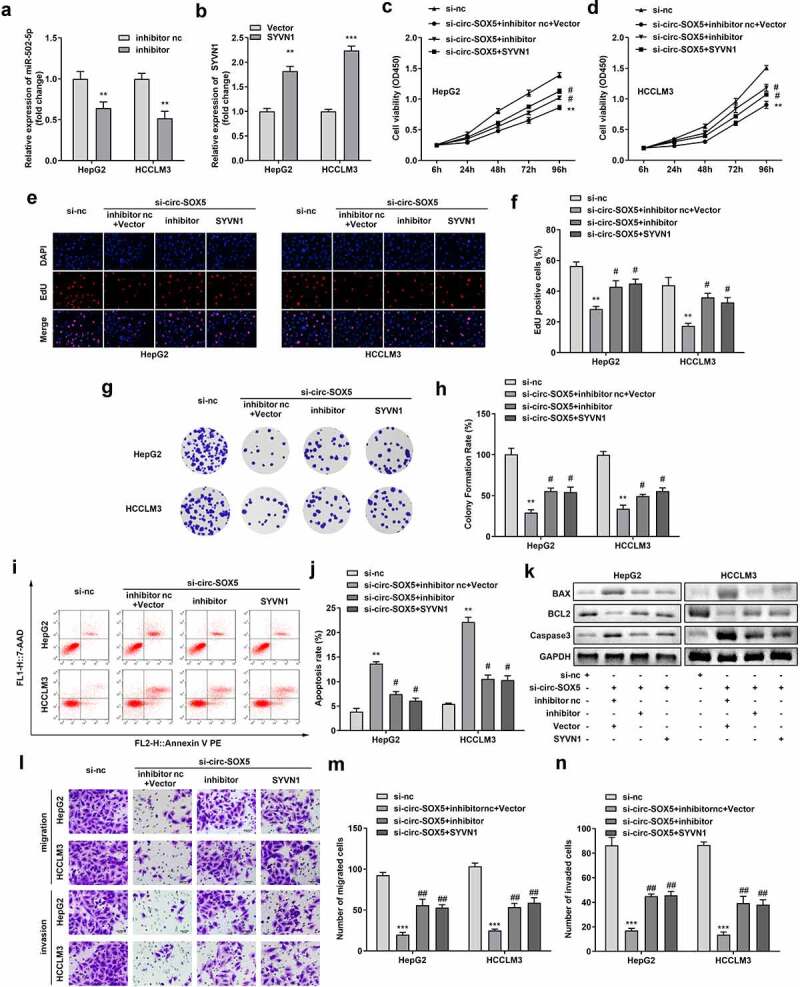
**MiR-502-5p inhibition and SYVN1 overexpression weakened the effects of circ-SOX5 knockdown on HCC cell**. (a) Inhibition of miR-502-5p in HCC cells. (b) Overexpression of SYVN1 in HCC cells. (c-d) Cell viability of HepG2 and HCCLM3 cells. (e-f) Cell proliferation of HCC cells. (g-h) Clone formation of HepG2 and HCCLM3 cells. (i-j) Quantity of cell apoptosis. (k) Expression of BAX, BCL2 and Caspase3. (l-n) The migration and invasion of the HCC cells, **P < 0.01, *** P < 0.001, # P < 0.05 versus control.

To further verify the potential of circ-SOX5 in HCC, rescue assays were performed. In Figure 5(a), miR-502-5p expression was significantly decreased in the miR-502-5p inhibitor group. SYVN1 was markedly upregulated in SYVN1 overexpression plasmids (Figure 5(b)). Both mir-502-5p inhibition and SYVN1 overexpression remarkably improved cell viability, proliferation, and clone formation in HCC cells (Figure 5(c-h)). Meanwhile, the apoptosis was significantly reduced after inhibition of miR-502-5p or overexpression of SYVN1 (Figure 5(i-j)). Moreover, downregulation of miR-502-5p or upregulation of SYVN1 abrogated the effects of circ-SOX5 knockdown on the expression of BAX, caspase-3, and BCL2 (Figure 5(k)). Besides, both mir-502-5p inhibition and SYVN1 overexpression remarkably improved cell migration and invasion in HCC cells (Figure 5(l-n)).

## 4. Discussion

HCC has poor prognosis and high recurrence rate, which poses a huge threat to human health [32]. In this study, we investigated the role of circ-SOX5 in HCC. However, circ-SOX5 knockdown increased apoptosis and suppressed the proliferation of HCC cells. Moreover, circ-SOX5 sponges miR-502-5p to upregulate SYVN1. Therefore, the circ-SOX5/miR-502-5p/SYVN1 axis may be a potential therapeutic target for HCC.

Various differently expressed genes or circRNAs participated in the HCC progressions via regulating the cell biological behaviors. Bian et al. [33] demonstrated that YTHDF1 was also a potential molecular target for HCC treatment, which promoted the aggressive phenotypes by facilitating epithelial-mesenchymal transition. Besides, dysregulated circRNAs are associated with the development of HCC. For instance, overexpression of circ-CRIM1 promotes the proliferation and angiogenesis of HCC [34]. However, hsa_circ_0007059 inhibits HCC growth and stemness [35]. These findings indicate that circRNAs may function as oncogenes or tumor suppressors in HCC. Therefore, identifying the exact role of circRNAs in HCC is vital. In this study, circ-SOX5 was overexpressed in HCC cells. circ-SOX5 knockdown suppressed the proliferation and promoted the apoptosis of HCC cells, suggesting that circ-SOX5 may function as an oncogene in HCC.

circRNAs function as ceRNAs to participate in biological processes by sponging miRNA(s). After confirming the regulatory effect of circ-SOX5 on HCC, we predicted the target genes downstream of circ-SOX5 and found that miR-502-5p interacts with circ-SOX5. miR-502-5p can regulate renal cell carcinoma [36], ovarian cancer [37], gastric cancer [34], and other cancers, but its association with HCC has not yet been reported. In this study, we found that miR-502-5p expression in liver tumor cells was reduced, and the inhibition of miR-502-5p expression weakened the effect of circ-SOX5 knockdown on tumor cells, indicating that miR-502-5p plays an important role in the regulatory axis of circ-SOX5.

Synovialis (SYVN1) is a target gene of miR-502-5p predicted by bioinformatics methods [38,39]. Some studies have shown that SYVN1 is related to the development of various tumors [35,40], and SYVN1 promotes the proliferation of tumor cells in HCC [41]. We also confirmed that high expression of SYVN1 could enhance the viability, proliferation, and clone formation of HCC cells, as well as affect the regulation of circ-SOX5.

## 5. Conclusion

In summary, HCC cells showed increased expression of circ-SOX5. Inhibition of circ-SOX5 increased the apoptosis of tumor cells and reduced the proliferation of tumor cells through miR-502-5p/SYVN1 axis. These findings may provide potential therapeutic targets or diagnostic markers for the treatment of HCC.

## Highlights

1. Circ-SOX5 expressed highly in HCC.
2. Circ-SOX5 directly targeted miR-502-5p
3. SOX5/miR-502-5p/SYVN1 axis promotes the development of HCC
